# High-T_g_ Polyimide Matrix Composites via Backbone Ethynyl Crosslinking: Preparation and Short-Term High-Temperature Performance

**DOI:** 10.3390/polym18091016

**Published:** 2026-04-22

**Authors:** Jinsong Sun, Chengyu Huang, Shengxiong Li, Hansong Liu, Lei Yao, Peng Zhang, Xiangyu Zhong, Jianwen Bao

**Affiliations:** 1 AVIC Manufacturing Technology Institute Composite Technology Center, Beijing 101300, China; sunjsbuaa@163.com (J.S.); huangchengyu@buaa.edu.cn (C.H.); liuhansongzhfc@foxmail.com (H.L.); 1570845448@163.com (L.Y.); xyzhong2003@sohu.com (X.Z.); 2 AVIC Composite Corporation Ltd., Beijing 101300, China; lisx057@avic.com

**Keywords:** polyimide resin, backbone ethynyl crosslinking, carbon fiber composites, high-temperature mechanical properties

## Abstract

Carbon fiber-reinforced polyimide composites are critical for aerospace applications in high-temperature environments of 300–500 °C. However, conventional PMR-15- and PEPA-terminated polyimides are limited by their insufficient glass transition temperatures (T_g_) and low crosslinking densities. This study proposes a reactive backbone construction strategy by employing 4,4′-(ethyne-1,2-diyl)diphthalic anhydride (EBPA) as a difunctional monomer copolymerized with asymmetric 2,3,3′,4′-biphenyl tetracarboxylic dianhydride (α-BPDA) and 4,4′-oxydianiline to synthesize polyimide resins containing both backbone ethynyl and terminal phenylethynyl groups. The effects of EBPA content on the curing behavior, thermomechanical properties, and elevated temperature mechanical performance were systematically investigated. The incorporation of EBPA significantly elevated T_g_ from 378 °C to 486 °C. Compared to the EBPA-0 control, the optimized EBPA-2 composite exhibited 7.3% and 3.6% improvements in room temperature flexural strength and modulus, respectively. Notably, at 400 °C, EBPA-2 demonstrated retention rates of 69.9%, 93.7%, and 61.6% for flexural strength, flexural modulus, and interlaminar shear strength, exceeding EBPA-0 by 16.9, 8.9, and 18.6 percentage points. SEM analysis confirmed the effective suppression of interfacial debonding at elevated temperatures. These findings elucidate the structure–property relationships between molecular structure, T_g_, and short-term high-temperature mechanical retention, providing a promising resin matrix for advanced aerospace carbon fiber composites.

## 1. Introduction

With the rapid development of aerospace vehicles and advanced aero-engines, the demand for carbon fiber-reinforced polymer matrix composites (CFRP) capable of service in high-temperature environments of 300–500 °C has intensified significantly [1,2,3]. Thermosetting polyimide resins are primary candidates for heat-resistant matrices; however, their glass transition temperatures (T_g_) and retention of mechanical properties at elevated temperatures remain critical limitations restricting the expansion of CFRP application ranges [4,5,6]. Although the traditional norbornene-terminated polyimide PMR-15 boasts mature processing and widespread application, it still possesses inherent limitations. The T_g_ of PMR-15 is only 340 °C, beyond which the modulus drops sharply due to enhanced segmental mobility. Meanwhile, the methylene (-CH_2_-) groups and aliphatic norbornene end groups are prone to degradation, so the PMR-15 composites could only be applied in service conditions below 315 °C [7,8,9,10]. Alston et al. [11] demonstrate that the thermal stability issues of PMR-15 resin originate from intrinsic chemical structure defects that cannot be fundamentally resolved through process optimization. While the second-generation PMR-II elevated T_g_ to 390 °C by adjusting monomer ratios, the practical application has been restricted by compromised processability and increased brittleness [12,13].

To overcome the limitations of PMR resins, phenylethynyl groups have been employed as end-capping agents to prepare phenylethynyl-terminated polyimides, which elevate the T_g_ above 371 °C through aromatic crosslinking reactions and significantly improve thermal stability [14,15,16,17,18]. Hergenrother et al. [19] utilized 4-phenylethynylphthalic anhydride (4-PEPA) as an end-capping agent, yielding resins with excellent high-temperature mechanical properties under 350 °C. Connell et al. [20] report PETI-298 and PETI-330 resins employing asymmetric 2,3,3′,4′-biphenyl tetracarboxylic dianhydride (α-BPDA), introducing non-coplanar isomeric aromatic structures into the molecular framework to improve solubility and processability while maintaining high T_g_. However, the crosslinking density afforded by monofunctional end groups is limited, typically resulting in flexural strength retention below 60% at 400 °C. Yokota et al. [21,22] systematically investigated asymmetric polyimides containing α-BPDA and found that rigid asymmetric structures could increase interchain spacing and suppress chain entanglement, thereby improving solubility while enhancing T_g_. The Triple-A PI composite exhibits excellent mechanical property retention at 350 °C. It is evident that the introduction of α-BPDA with a non-coplanar structure enables the synergistic improvement of processability and heat resistance.

Introducing ethynyl groups into the polyimide backbone is an effective strategy to enhance crosslinking density, and 4,4′-(ethyne-1,2-diyl)diphthalic anhydride (EBPA) has attracted considerable attention as a difunctional monomer [23,24]. This monomer simultaneously contains reactive anhydride groups and functional ethynyl groups, wherein the anhydride groups can condense with diamines to form the polyimide backbone, enabling uniform distribution of ethynyl groups throughout the molecular skeleton. Researchers have incorporated EBPA into thermoplastic polyimide molecular structures to prepare films, improving film thermal stability through intermolecular chain crosslinking via reactive ethynyl structures [25,26,27]. Compared with end-capped ethynyl groups, backbone ethynyl groups can form more perfect three-dimensional crosslinking networks, significantly enhancing T_g_ and high-temperature creep resistance. Fernberg et al. [28,29,30] introduced ethynyl dianhydride into the main chain to develop MHT-R polyimide resin suitable for RTM molding. This resin exhibits melt viscosity of only 0.2–2.0 Pa·s in the range of 250–320 °C, with a cured T_g_ reaching 460 °C, and carbon fiber-reinforced composites could withstand high temperatures of 350–400 °C. However, MHT-R resin is based on a 6FDA structure. Although the introduction of fluorine atoms improves solubility, it reduces high-temperature modulus and dimensional stability, and the cost is relatively high.

However, existing research on EBPA-based polyimide resins has primarily focused on RTM-process polyimide composites [28,29] and resin-curing mechanisms [31,32], while systematic investigations on the high-temperature mechanical properties of autoclave-molded composites and the performance retention mechanisms under load-bearing environments above 400 °C remain scarce. Furthermore, the synergistic effects between EBPA and rigid asymmetric dianhydrides (such as α-BPDA) and their influence on processing performance still require exploration. This study proposes a reactive backbone construction strategy, employing EBPA as a difunctional monomer to copolymerize with α-BPDA and 4,4′-oxydianiline (4,4′-ODA) to synthesize a series of polyimide resins containing both backbone ethynyl and terminal phenylethynyl groups. By systematically regulating EBPA content, the effects on curing behavior, thermomechanical properties, and short-term high-temperature mechanical properties at 350–450 °C were investigated. The structure–property relationships among molecular structure, T_g_, and high-temperature performance retention were elucidated, providing theoretical and experimental foundations for the development of carbon fiber-reinforced polyimide composites suitable for service in elevated temperature environments.

## 2. Materials and Methods

### 2.1. Materials

T800-grade carbon fiber U-8190 plain weave fabric, with an areal density of 190 g/m^2^, was provided by Weihai Tuozhan Fiber Co., Ltd., Weihai, China. The fabric was subjected to thermal desizing treatment at 350 °C. 4-phenylethynylphthalic anhydride (4-PEPA), 2,3,3′,4′-biphenyl tetracarboxylic dianhydride (α-BPDA) were procured from Changzhou Sunshine Pharmaceutical Co., Ltd. (Changzhou, China). 4,4′-(ethyne-1,2-diyl)diphthalic anhydride (EBPA) was supplied by TCI (Shanghai) Development Co., Ltd. (Shanghai, China). 4,4′-Oxydianiline (4,4′-ODA) was provided by China Tech (Tianjin) Chemical Co., Ltd., (Tianjin, China) and N,N-dimethylacetamide (DMAc) of analytical reagent (AR) grade was purchased from Sinopharm Chemical Reagent Co., Ltd. (Shanghai, China). All reagents were used as received without further purification.

### 2.2. Synthesis of EBPA Polyimide Resin

**Figure 1:**
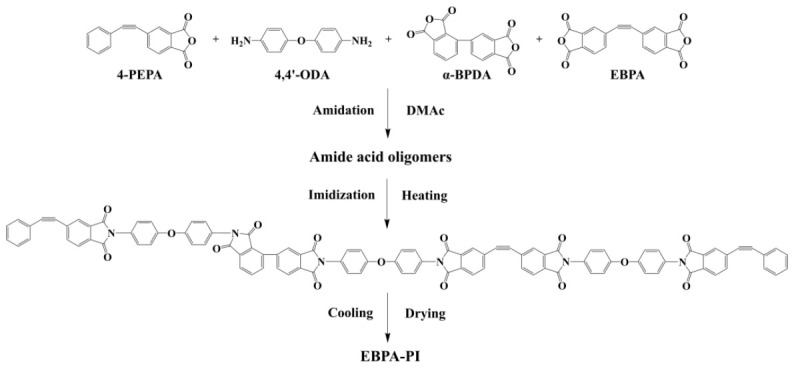
Synthesis route of EBPA-based polyimide resin.

Into a dried three-neck round-bottom flask equipped with a mechanical stirrer, high-purity nitrogen inlet, and a reflux condenser, 4,4′-ODA was dissolved in DMAc. The mixture was stirred under a nitrogen atmosphere until a homogeneous and transparent diamine solution was obtained. Subsequently, stoichiometric amounts of dianhydride monomers EBPA and α-BPDA were weighed, then dissolved in DMAc to prepare the dianhydride solution. The dianhydride solution was added dropwise to the diamine solution under continuous stirring. After reaction at room temperature for 2 h, 4-PEPA pre-dissolved in DMAc was introduced as an end-capping agent. The mixture was mechanically stirred at 75 °C for 1 h until a transparent poly(amic acid) (PAA) solution was formed. Upon cooling, the PAA solution was subjected to rotary evaporation at 160 °C to remove the solvent. The resulting solid powder was thermally treated at 230 °C under vacuum for 2 h to obtain the EBPA-based polyimide resin powder for physicochemical characterization. Samples for TGA required further curing at 385 °C for another 2 h. Five resins with varying compositions were synthesized by adjusting the molar ratio of dianhydride monomers (EBPA and α-BPDA) to diamine monomer (4,4′-ODA), named as EBPA-0, EBPA-1, EBPA-2, EBPA-3, and EBPA-4, respectively. The synthetic route of EBPA-based polyimide resin is illustrated in Figure 1, and detailed feed ratios are summarized in Table 1.

### 2.3. Fabrication of EBPA Polyimide-Based Composites

The obtained PAA solution was uniformly impregnated onto U-8190 carbon fiber fabric via hand lay-up/brush impregnation to prepare carbon fiber-reinforced polyimide wet prepreg, with a resin mass fraction of 45%. The solvent-removed wet prepreg was cut into 330 mm × 250 mm plies and stacked according to the [0]_10_ lay-up sequence. After pre-treatment to remove residual solvent, the laminate was transferred into an autoclave and processed following the cure cycle of 210 °C/60 min + 280 °C/30 min + 350 °C/60 min + 385 °C/300 min, with 1.5 MPa pressure application at 280 °C. The theoretical fiber volume fraction (V_f_) of the composite was 53.4%. The fabricated composite laminates were subjected to non-destructive testing (NDT) and subsequently machined into mechanical test specimens for flexural and interlaminar shear strength characterization. The actual fiber volume fraction was shown in Table S1 (Supplementary Materials), and the NDT results are presented in Figure S1.

### 2.4. Characterization

#### 2.4.1. General Characterizations

The surface and cross-sectional morphologies of the samples were examined using a Quanta 450 FEG field-emission environmental scanning electron microscope (FEI Company, Hillsboro, OR, USA), operated at an accelerating voltage of 10.0 kV. Wide-angle X-ray diffraction (XRD) measurements were performed on a D/MAX 2500 X-ray diffractometer (Rigaku Company, Tokyo, Japan) in continuous scanning mode, 2θ ranging from 5° to 70°. Fourier-transform infrared (FTIR) spectroscopy was performed on a FTIR 750 spectrometer (Nicolet Company, Madison, WI, USA) using KBr pellets between 4000 cm^−1^ and 500 cm^−1^ in air.

#### 2.4.2. Thermal Analysis

Thermogravimetric analysis (TGA) was conducted on a STA 499 F3 simultaneous thermal analyzer (Netzsch, Selb, Germany) to evaluate the thermal stability of the cured polyimides. Measurements were performed under a nitrogen atmosphere at a heating rate of 10 °C/min from room temperature (RT) to 800 °C. Differential scanning calorimetry (DSC) was carried out using a Q20 differential scanning calorimeter (TA Instruments, New Castle, DE, USA) to investigate the curing and cross-linking behavior of the resins. The samples were heated from RT to 500 °C at 10 °C/min under nitrogen protection. Dynamic mechanical analysis (DMA) was performed on a Q800 dynamic mechanical analyzer (TA Instruments, USA) to determine the glass transition temperature (T_g_) of the cured resins. The measurements were conducted according to the ASTM D7028 standard [33] using a dual-cantilever clamp mode with specimen dimensions of 60 mm × 10 mm × 2 mm. The temperature was ramped from RT to 550 °C at a heating rate of 5 °C/min. Rheological properties were measured using an AR 2000 rotational rheometer (TA Instruments, USA) equipped with parallel-plate fixtures. Prior to testing, the polyimide resin powders were cold-pressed into circular disks with a diameter of approximately 25 mm and a thickness of 1–2 mm using a dedicated mold. The tests were performed from RT to 400 °C at a heating rate of 2 °C/min.

#### 2.4.3. Mechanical Characterization

Interlaminar shear strength (ILSS) of carbon fiber-reinforced polyimide composites was measured using an INSTRON 5982 universal testing machine (Instron Corporation, Norwood, MA, USA) in accordance with the ASTM D 2344 standard [34]. Specimens with dimensions of 20 mm × 6 mm × 4 mm were loaded in short-beam shear mode. The ILSS was calculated according to Equation (1):

(1) $$ILSS=\frac{3F}{4bh}$$

where *F* is the maximum applied load (N), *b* is the specimen width (mm), and *h* is the specimen thickness (mm). A minimum of six valid measurements were obtained for each sample group, with the average values and standard deviations reported.

Flexural properties were evaluated on the same testing machine following the ASTM D 790 standard [35] using a three-point bending mode. The specimen dimensions were 160 mm × 12 mm × 2 mm with a span-to-thickness ratio of 60. The flexural strength (*σ_f_*) was determined using Equation (2):

(2) $$\sigma_{f}=\frac{3PL}{2bh^{2}}$$

where *P* is the load at fracture (N), *L* is the support span (mm), *b* is the specimen width (mm), and *h* is the specimen thickness (mm). A minimum of six valid measurements were obtained for each sample group, the average values and standard deviations were reported. For high-temperature mechanical tests, the specimens were heated to specific temperatures in the testing chamber and held for 5 min.

## 3. Results and Discussion

### 3.1. Curing Behavior and Structure Analysis of EBPA-Based Polyimide Resin

**Figure 2:**
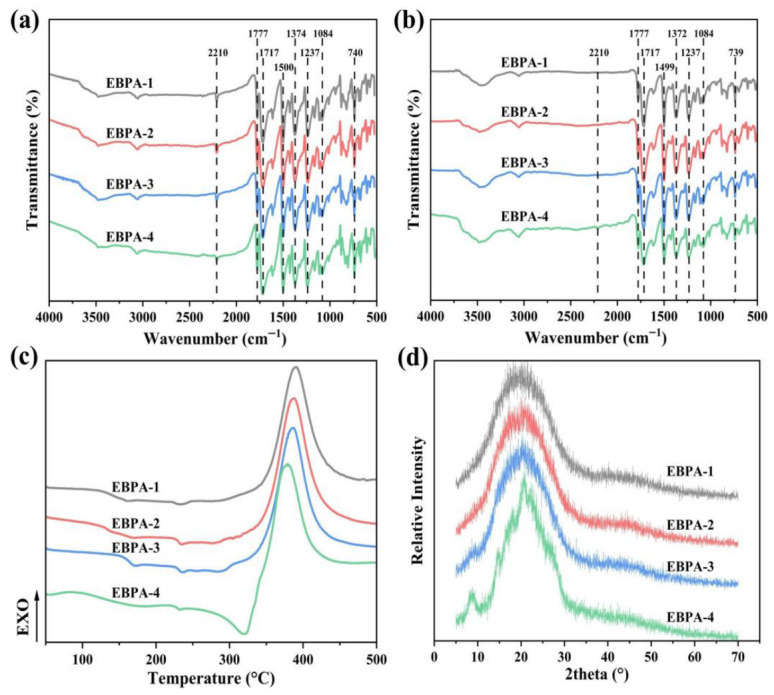
Structural analysis of EBPA-based polyimide: (**a**) Infrared spectrum after heat treatment at 230 °C; (**b**) Infrared spectrum after heat treatment at 385 °C; (**c**) DSC curves and (**d**) XRD curves.

The synthesis of polyimide resin involves three sequential reaction stages: amidation (formation of polyamic acid), imidization (cyclization to imide rings), and curing (cross-linking to form network structures). Fourier-transform infrared spectroscopy (FT-IR) was employed to qualitatively analyze the EBPA-based polyimide resins treated at various temperatures, aiming to identify the molecular structure types. Figure 2a presents the FT-IR spectra of polyimide resins with different EBPA contents after thermal treatment at 230 °C. The characteristic absorption peaks observed at 1777 cm^−1^, 1717 cm^−1^, and 740 cm^−1^ are assigned to the asymmetric stretching, symmetric stretching, and bending vibrations of the carbonyl groups (C=O) in the imide rings, respectively. The band at 1374 cm^−1^ corresponds to the C-N stretching vibration within the imide rings. It could be observed that no O-H stretching vibration absorption bands were detected in the range of 3650–3200 cm^−1^ for all resin formulations, indicating complete imidization and the formation of typical imide ring structures upon thermal treatment.

Additionally, characteristic absorption peaks arising from benzene ring skeletal vibrations were observed at 1500 cm^−1^ and 1084 cm^−1^. Of particular note was the stretching vibration band of the alkyne groups (C≡C) detected near 2210 cm^−1^, which originated from the ethynyl moieties present in both 4-PEPA and EBPA molecular structures. The persistence of this peak after thermal treatment at 230 °C indicated that the curing and cross-linking reactions had not yet been initiated in the polyimide resins at this stage.

Figure 2b displays the FT-IR spectra of polyimide resins with varying EBPA contents following thermal treatment at 385 °C. In comparison with the spectra obtained after imidization at 230 °C, the C≡C triple-bond stretching vibration band at 2210 cm^−1^ had essentially disappeared for all formulations, with only a weak residual absorption detected in the EBPA-4 curve. The intensities of all other characteristic bands remained essentially unchanged. This observation demonstrates that the ethynyl groups in the molecular chains of three resin formulations had undergone nearly complete cross-linking reactions upon thermal treatment at 385 °C, whereas EBPA-4 retained a minor fraction of unreacted alkyne moieties.

Figure 2c presents the DSC curves of polyimide resins with varying EBPA contents. It was evident that EBPA-4 exhibited a distinct endothermic peak around 320 °C, which was attributed to the absence of α-BPDA in the dianhydride composition, resulting in relatively higher molecular symmetry and consequently an elevated oligomer melting point. This conclusion was further corroborated by XRD analysis (Figure 2d), compared with the EBPA-1, EBPA-2 and EBPA-3 resins, EBPA-4 showed relatively sharp diffraction peaks at 2θ ≈ 10° and 20°, indicating distinctive crystalline characteristics and thus a higher melting point.

The characteristic reaction temperatures and enthalpy values of the polyimide resins are summarized in Table 2. With increasing EBPA content in the dianhydride composition, the ethynyl group content in the oligomer increased, leading to enhanced probability of the ethynyl moieties during the cross-linking reactions. This was manifested by a gradual decrease in both the onset and peak cross-linking temperatures, accompanied by a significant increase in cross-linking enthalpy. In the DSC curves, this trend was reflected by a shift to lower temperatures of the exothermic cross-linking peak with increasing EBPA content.

### 3.2. Thermal and Process Properties Analysis of EBPA-Based Polyimide Resin

**Figure 3:**
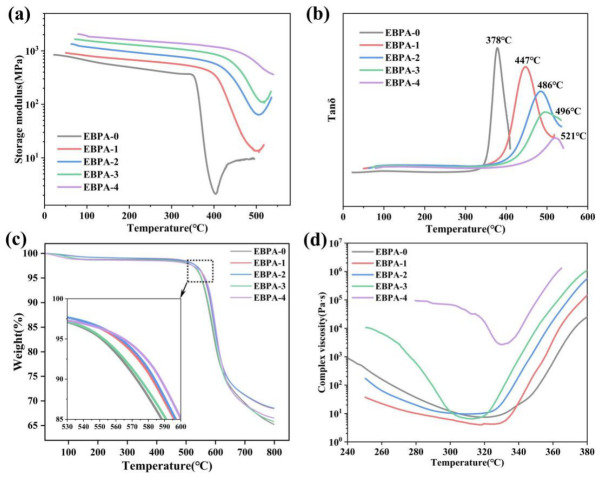
Thermal and processability analysis of EBPA-based polyimides: (**a**) Storage modulus curves of DMA; (**b**) Tanδ curves of DMA; (**c**) TGA curves and (**d**) Rheology curves.

The prepared polyimide oligomer powders were placed into a molding cavity with dimensions of 80 mm × 80 mm × 6 mm and heated using a hot press. When the temperature reached 300 °C, a pressure of 5 MPa was applied. Subsequently, the temperature was further increased to 385 °C and held for 2 h to complete the curing reaction. After cooling and demolding, polyimide castings were obtained. The thermomechanical properties of polyimide resins with varying EBPA contents were characterized by dynamic mechanical analysis (DMA) (Figure 3a,b), and the relevant data are summarized in Table 3.

The DMA results revealed that the polyimide resin without EBPA exhibited a glass transition temperature (T_g_) of merely 378 °C. Increasing the EBPA content in the dianhydride composition to 30%, the T_g_ was significantly elevated to 447 °C. Both the storage modulus and T_g_ demonstrated a monotonically increasing trend with further rising EBPA proportion. These phenomena were attributed to the formation of additional cross-linking sites by the ethynyl groups introduced via EBPA during the curing process, which led to increased cross-linking density, decreased molecular weight between cross-links, and enhanced chain rigidity, thereby resulting in higher T_g_ values. Meanwhile, the constraining effect of the cross-linked network on segmental mobility was intensified, which was manifested by a gradual decrease in the loss factor (tan δ) peak intensity with increasing EBPA content.

Thermogravimetric analysis (TGA) was employed to characterize the thermal decomposition temperatures of the polyimide resins. Figure 3c presents the TGA curves of the five polyimide resins measured under a nitrogen atmosphere at a heating rate of 10 °C/min. Data analysis revealed that polyimide resins synthesized from dianhydride mixtures with varying EBPA contents all exhibit excellent thermal stability. The 5% weight loss temperatures (T_d5%_) of resins with different structures range from 561 °C to 565 °C, showing no significant correlation with EBPA proportion.

Rotational rheometry was employed to characterize the viscosity–temperature behavior of polyimide resins with varying EBPA contents. Figure 3d presents the rheological curves of the oligomers for each resin formulation. The results indicate that the minimum viscosities of EBPA-0, EBPA-1, EBPA-2, and EBPA-3 were 7.41 Pa·s, 3.98 Pa·s, 9.61 Pa·s, and 6.44 Pa·s, respectively, whereas EBPA-4 exhibited a substantially higher minimum viscosity of 2684 Pa·s. The dianhydride compositions of EBPA-0 to EBPA-3 all contained α-BPDA, whose rigid and asymmetric molecular structure increased the interchain spacing and effectively suppressed chain entanglement, resulting in significantly reduced melt viscosity and a broad processing window with favorable processability. In contrast, EBPA-4 showed negligible viscosity change during the initial heating stage, which was attributed to the crystalline state of the oligomer at this phase. The excessive viscosity prevented adequate contact with the testing platform. As the temperature increased, the crystalline domains gradually melted, the viscosity decreased accordingly, and sufficient contact between the oligomer and the testing platform was established, ultimately exhibiting a characteristic viscosity–temperature profile with an initial decrease followed by a subsequent increase.

### 3.3. High-Temperature Mechanical Properties of EBPA Polyimide-Based Composites

**Figure 4:**
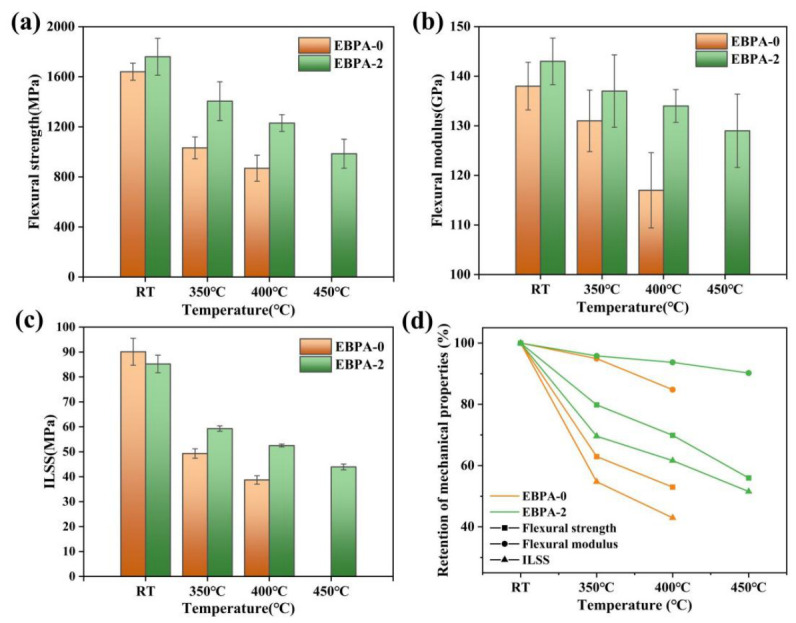
The high-temperature performance of composites: (**a**) flexural strength, (**b**) flexural modulus, (**c**) ILSS and (**d**) retention of mechanical properties at elevated temperatures.

For autoclave molding applications, adequate resin fluidity (typically <100 Pa·s, optimally 1–10 Pa·s) and a broad low-viscosity processing window are essential to ensure complete fiber impregnation and proper laminate consolidation. Considering the thermal and rheological characteristics of EBPA-based polyimide resins, both EBPA-2 and EBPA-3 resins exhibited elevated glass transition temperatures coupled with minimum viscosities below 10 Pa·s. Specifically, EBPA-2 achieved an optimal balance among minimum melt viscosity, processing window and T_g_, whereas EBPA-3 showed a narrower processing window of 42 °C, despite its higher T_g_. Therefore, EBPA-2 was selected as the matrix for carbon fiber-reinforced composite fabrication, with EBPA-0 serving as the control matrix. The high-temperature mechanical properties of both composites were systematically investigated at various temperatures, as presented in Figure 4.

Both composites exhibited excellent mechanical properties at room temperature. Compared with EBPA-0 (flexural strength of 1640 MPa and flexural modulus of 138 GPa), EBPA-2 demonstrated increased flexural strength and modulus of 1760 MPa and 143 GPa, respectively, representing improvements of 7.3% and 3.6%. This confirms that the high crosslinking density of the EBPA structure could enhance composite stiffness. However, the ILSS of EBPA-2 was 85.2 MPa, 5.4% lower than that of EBPA-0 (90.1 MPa). This reduction could be attributed to the influence of the highly cross-linked network on the fiber–resin interface of EBPA-2 composite.

At 350 °C, EBPA-0 experienced significant performance degradation, with flexural strength, flexural modulus, and ILSS decreasing to 1032 MPa, 131 GPa, and 49.3 MPa, respectively, corresponding to retention rates of 62.9%, 94.9%, and 54.7%. At this temperature, which approached the glass transition temperature of EBPA-0 resin (378 °C), enhanced segmental mobility resulted in drastic deterioration of interfacial adhesion. In contrast, EBPA-2 maintained superior performance at 350 °C, achieving flexural strength, flexural modulus, and ILSS of 1405 MPa, 137 GPa, and 59.3 MPa, with retention rates of 79.8%, 95.8%, and 69.6%. When the test temperature was elevated to 400 °C, the performance gap between the two composites widened substantially. EBPA-0 underwent comprehensive mechanical deterioration, with flexural strength, flexural modulus, and ILSS plummeting to 869 MPa, 117 GPa, and 38.7 MPa, retention rates of merely 53.0%, 84.8%, and 43.0%. At this temperature, 22 °C above its T_g_, the resin matrix transitioned into the rubbery state with substantial rigidity loss. Conversely, EBPA-2 sustained appreciable performance levels at 400 °C, with flexural strength, flexural modulus, and ILSS of 1230 MPa, 134 GPa, and 52.5 MPa, corresponding to retention rates of 69.9%, 93.7%, and 61.6%. Compared with EBPA-0, EBPA-2 exhibited superior retention rates, with improvements of 16.9 percentage points for flexural strength, 18.6 percentage points for ILSS, and 8.9 percentage points for flexural modulus, demonstrating the performance superiority of the high-T_g_ matrix in high-temperature environments.

Furthermore, the EBPA-2 composite demonstrated considerable mechanical integrity at 450 °C, with flexural strength, flexural modulus, and ILSS of 985 MPa, 129 GPa, and 43.9 MPa, respectively, retention rates of 56.0%, 90.2%, and 51.5%. This exceptional performance was attributed to the significant elevation of resin T_g_ to 486 °C (an increase of 108 °C) through EBPA incorporation, ensuring that the matrix remained in a glassy state at 450 °C and effectively suppressed molecular segmental motion. The increased cross-linking density also contributed by restricting plastic deformation of the resin matrix and mitigated interfacial debonding and microcrack nucleation at elevated temperatures, thereby preserving the load-bearing capability of the composite.

**Figure 5:**
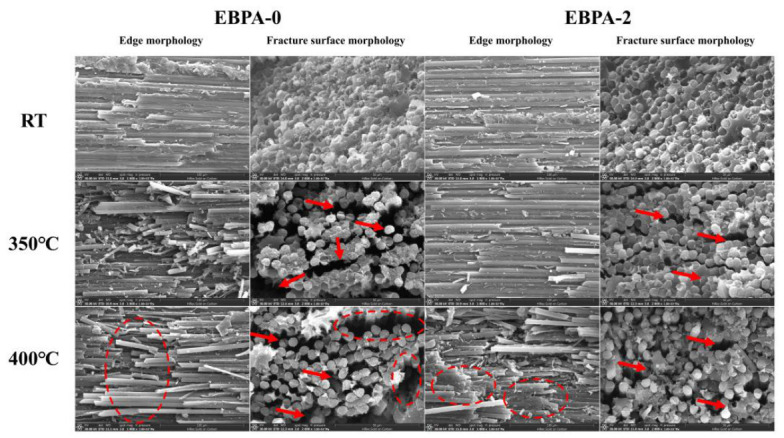
SEM images of composite fracture surfaces after ILSS and flexural tests.

The fracture morphologies of interlaminar shear specimens (side view) and flexural specimens (fracture surface) for EBPA-0 and EBPA-2 polyimide composite laminates tested at various temperatures were systematically examined by scanning electron microscopy (SEM), as presented in Figure 5. During interlaminar shear and flexural testing, the specimens were subjected to a complex stress state comprising interlaminar shear stress, flexural stress, transverse shear stress, and localized compressive stress. SEM observation of room-temperature-tested samples reveals that the fiber surfaces in both composite systems were uniformly coated with the resin matrix, with the interfacial regions remaining intact after mechanical failure, and no obvious fiber–matrix interfacial debonding was observed. The fracture surfaces of flexural specimens were relatively flat, exhibiting characteristic brittle fracture features. These results demonstrate that the resin matrix maintained favorable process compatibility with carbon fibers upon incorporation of EBPA monomers.

After testing at 350 °C, partial resin coating was still observable on the carbon fiber surfaces of the EBPA-2 composite, with resin bridging between adjacent fibers. SEM examination of flexural fracture surfaces indicated that microcracks initiated within the composite and propagated through the resin matrix, with the failure mode remaining predominantly brittle. However, the mechanical properties deteriorated due to defect nucleation. In contrast, EBPA-0 composites exhibited a significant transition in the failure mode, the test temperature approached the glass transition temperature of the resin matrix, intensifying molecular segmental motion and increasing resin fluidity. Under mechanical loading, crack initiation and propagation preferentially occurred at resin-starved regions on carbon fibers, ultimately leading to catastrophic failure. SEM analysis of flexural fracture surfaces revealed extensive void formation at fiber–matrix interfaces, with fiber fracture predominantly initiating at the loading points. The failure mode thus transformed from brittle fracture to loading-point damage-induced crack propagation.

At an elevated test temperature of 400 °C, SEM observation of EBPA-0 composite edges showed almost completely exposed fibers, with fracture surfaces exhibiting prominent fiber bundle pull-out and matrix detachment. Internal defects became substantially more pronounced, with fibers prone to fracture at weak locations accompanied by rapid crack extension associated with resin flow, resulting in significantly reduced transverse shear resistance. As the test temperature exceeded the glass transition temperature of the resin, the matrix rigidity decreased markedly with consequent plastic deformation, leading to drastic deterioration in mechanical properties. By comparison, EBPA-2 composites, benefiting from enhanced glass transition temperature through EBPA incorporation, maintained favorable fiber–matrix interfacial bonding at 400 °C. Side-view and fracture surface SEM observations revealed localized resin coating on carbon fiber surfaces with limited defect proliferation, reflecting enhanced resistance to fracture and effective suppression of transverse crack propagation from loading points through the thickness direction. Consequently, the retentions of high-temperature mechanical properties were significantly superior to that of the EBPA-0 composite.

## 4. Conclusions

In this study, a series of polyimide resins containing 4,4′-(ethyne-1,2-diyl)diphthalic anhydride (EBPA) were designed and synthesized. With increasing EBPA content, the onset and peak temperatures of cross-linking decreased while the reaction enthalpy increased, indicating enhanced cross-linking probability associated with elevated ethynyl concentrations. DMA results demonstrate that EBPA incorporation significantly elevated the glass transition temperature from 378 °C (EBPA-0) to 486 °C (EBPA-2), an increase of 108 °C, with a monotonic upward trend as EBPA content increased. This was attributed to the ethynyl cross-linking structure, which increased cross-linking density, decreased average molecular weight between cross-linking points, and enhanced chain rigidity. Compared with the EBPA-0, the EBPA-2 composite exhibited 7.3% and 3.6% improvements in flexural strength and modulus at room temperature. This study focuses on establishing baseline short-term mechanical retention as a prerequisite for subsequent long-term aging studies. At elevated temperatures, EBPA-2 demonstrated significant advantages. At 400 °C, the retention rates of flexural strength, flexural modulus, and interlaminar shear strength were 69.9%, 93.7%, and 61.6%, exceeding EBPA-0 by 16.9, 8.9, and 18.6 percentage points, respectively. SEM fractography confirmed that the high-T_g_ matrix effectively suppressed interfacial debonding and microcrack propagation at elevated temperatures. Therefore, this study demonstrates that a molecular structural design incorporating EBPA components significantly improves the retention of high-temperature mechanical properties for carbon fiber-reinforced composites across a broad temperature window. The novel EBPA-based polyimide resin, exhibiting excellent thermal resistance and processability, can serve as a resin matrix for high-temperature resistant composites, demonstrating substantial potential for applications in advanced manufacturing fields such as aerospace and aviation. Long-term thermo-oxidative aging, creep resistance, and thermal cycling behavior will be systematically investigated in future work to fully qualify these materials for extended service environments.

## Figures and Tables

**Table 1:** Molar ratios for EBPA-based polyimide resin synthesis.

Resin	Molar Ratios			
	4-PEPA	EBPA	α-BPDA	4,4′-ODA
EBPA-0	2	0	2	3
EBPA-1	2	0.6	1.4	3
EBPA-2	2	0.8	1.2	3
EBPA-3	2	1	1	3
EBPA-4	2	2	0	3

**Table 2:** Curing characteristic parameters of EBPA-based polyimides.

Resin	Content of EBPA (mol%)	Melting Point/°C	Onset/°C	Peak/°C	Enthalpy/(J/g)
EBPA-1	30%	232	352	390	174.37
EBPA-2	40%	234	350	388	184.01
EBPA-3	50%	236	347	386	195.14
EBPA-4	100%	234	339	378	222.13

**Table 3:** TGA and DMA data of EBPA-based polyimides.

Samples	Content of EBPA (mol%)	T_g_(E’)/°C	T_g_ (tanδ)/°C	T_d_ (onset)/°C	T_d5%_ (N_2_)/°C
EBPA-0	0%	352	378	549	561
EBPA-1	30%	403	447	549	561
EBPA-2	40%	428	486	550	563
EBPA-3	50%	445	496	551	563
EBPA-4	100%	466	521	560	565

## Data Availability

The original contributions presented in this study are included in the article/Supplementary Materials. Further inquiries can be directed to the corresponding authors.

## Supplementary Materials

The following supporting information can be downloaded at: https://www.mdpi.com/article/10.3390/polym18091016/s1, Table S1. Fiber volume fraction and porosity of laminates; Figure S1. Porosity testing and C-Scan images of laminates.
