# Supplementary material for: High-Tg Polyimide Matrix Composites via Backbone Ethynyl Crosslinking: Preparation and Short-Term High-Temperature Performance
Source: Polymers (Basel). 2026 Apr 22;18(9):1016. doi: 10.3390/polym18091016 (PMC13165319; doi:10.3390/polym18091016)
Supplement: Supplementary file 1 [file polymers-18-01016-s001.zip › polymers-4241095-supplementary.pdf]

Supplementary Information

In this study, carbon fiber reinforced EBPA-0 and EBPA-2 composite laminates were prepared. The actual fiber volume fraction was calculated from measured laminate thickness (Table S1). The sample integrity was evaluated by ultrasonic C-scan (NDT) and optical microscopy, and porosity characterization was adopted in accordance with GB/T 3365-2008, as shown in the Figure S1. No delamination or concentrated voids were detected.

Table S1. Fiber volume fraction and porosity of laminates.

Laminates	Thickness/mm	Average Thickness/mm	Actual Fiber Volume Fraction (V_f)/%	Porosity/%
EBPA-0	1.938<br>1.946<br>1.913	1.932	55.25	Not detected
EBPA-2	1.981<br>1.935<br>1.997	1.971	54.16	Not detected

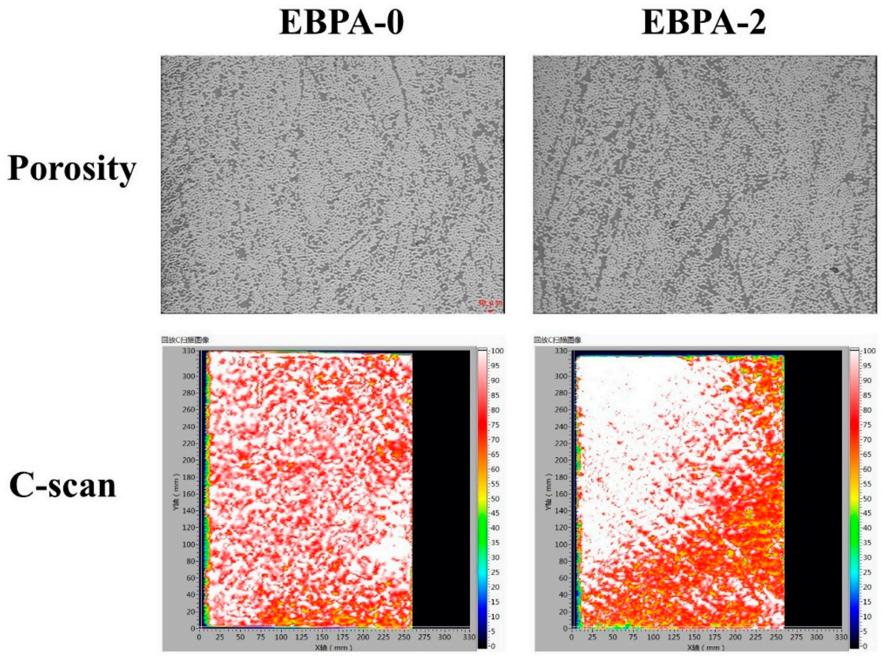

Figure S1. Porosity testing and C-Scan images of laminates.
